# Marine medicinal glycomics

**DOI:** 10.3389/fcimb.2014.00005

**Published:** 2014-01-29

**Authors:** Vitor H. Pomin

**Affiliations:** Program of Glycobiology, Institute of Medical Biochemistry Leopoldo de Meis, and University Hospital Clementino Fraga Filho, Federal University of Rio de JaneiroRio de Janeiro, Brazil

**Keywords:** carbohydrate-based drug development, chitin, chitosan, glycosaminoglycans, sulfated fucans, sulfated galactans

## Abstract

Glycomics is an international initiative aimed to understand the structure and function of the glycans from a given type of cell, tissue, organism, kingdom or even environment, as found under certain conditions. Glycomics is one of the latest areas of intense biological research. Glycans of marine sources are unique in terms of structure and function. They differ considerably from those of terrestrial origin. This review discusses the most known marine glycans of potential therapeutic properties. They are chitin, chitosan, and sulfated polysaccharides named glycosaminoglycans, sulfated fucans, and sulfated galactans. Their medical actions are very broad. When certain structural requirements are found, these glycans can exhibit beneficial effects in inflammation, coagulation, thrombosis, cancer growth/metastasis, and vascular biology. Both structure and therapeutic mechanisms of action of these marine glycans are discussed here in straight context with the current glycomic age through a project suggestively named marine medicinal glycomics.

## Introduction

After the launch of many international biological *ome* initiatives, the glycome has now emerged as a source of great information (Hart and Copeland, 2010). Glycome is the project and glycomics is the studies concerned with the science of carbohydrates or glycobiology. Glycomics aims to describe systematically and comparatively the specific or general properties of the carbohydrates. These carbohydrates may be within a repertoire of a given type of cell, tissue, organism, kingdom, or a certain environment as found under specific conditions. Glycomics is focused on the studies and description of the structural and biological functions of carbohydrates. The particular underlying mechanisms of sugar biosynthesis, catabolism, and the nature of molecular interactions with functional proteins involved in health and pathology are also relevant topics of study in glycomics.

Glycomics has brought more challenges than other *ome* projects. The reason is that carbohydrates are the utmost complex biomolecules in terms of structure. High dynamic behavior, conformational fluctuations, diversity of monomers, glycosidic linkages, enantiomers, anomericity, extensive and inhomogeneous post-polymerization modifications are all relevant contributors to greatly enhance structural complexity in glycobiology. Moreover, the number of carbohydrate classes is very high. They include *N*-linked or *O*-linked oligosaccharides in glycoproteins, glycosaminoglycans (GAGs) in proteoglycans, sulfated fucans (SFs), sulfated galactans (SGs) and many others. Because of this, glycomics is a sum of many individual subprojects rather than a single and unique project. This helps to decrease the complexity of the system. Based on this natural division new terminologies are being created to describe the subprojects. Some examples are sialome (for sialic acid-containing glycans) (Cohen and Varki, 2010), glycosaminoglycanome (for GAGs) (Gesslbauer and Kungl, 2006), heparanome (for heparan sulfate) (Lamanna et al., 2007), proteoglycanome (for proteoglycans) (Gesslbauer et al., 2007), fucanome (for SFs) (Pomin, 2012a,b), and galactanome (for SGs) (Pomin, 2012a,b).

The most medically relevant functions of carbohydrates are those related with clinical treatment (therapy) or prevention (prophylaxis). These areas of glycobiology are boosted not only to develop new health care products but due to the efforts of multinational pharmaceutical companies to design and manufacture novel carbohydrate-based drugs. Although several glycans have therapeutic properties those of marine origin have a special position. This is particularly due to the unique structural features that are not found in naturally occurring terrestrial sources. The medicinal mechanisms of action of the marine glycans are also quite distinct (Pomin and Mourão, 2008; Pomin, 2009). Research using structurally well-defined glycans from marine organisms helps to achieve accurate structure-function relationships (Pomin, 2012b,c). Marine sources are rich in glycans of well-defined chemical structures that can be used to achieve these accurate relationships, as discussed further. These accurate correlations between structure and medical function are extremely important for drug discovery and development, especially when novel glycans are under investigation.

This document aims to describe, in a systematic way, the main structural and medical properties of the most well known glycans from the sea. These glycans are chitin, chitosan, and sulfated polysaccharides (SPs), named GAGs, SFs, and SGs. When certain structural features are present, these glycans can exhibit beneficial activities in inflammation, coagulation, thrombosis, cancer, and vascular biology. The underlying mechanism of actions for their medical effects will be described here individually for each class of marine polysaccharide. All the background provided herein will be discussed in direct connection with glycomics. In fact, this set of information strongly supports the incorporation and development of a new subproject in glycomics, which is suggestively named *marine medicinal glycomics*. The objective of this subproject in the currently ongoing glycomic era is not limited to dissemination of knowledge regarding therapeutic marine carbohydrates but meant to assist research programs focused on marine carbohydrate-based drug discovery and development.

## Chitin and chitosan

**Figure 1:**
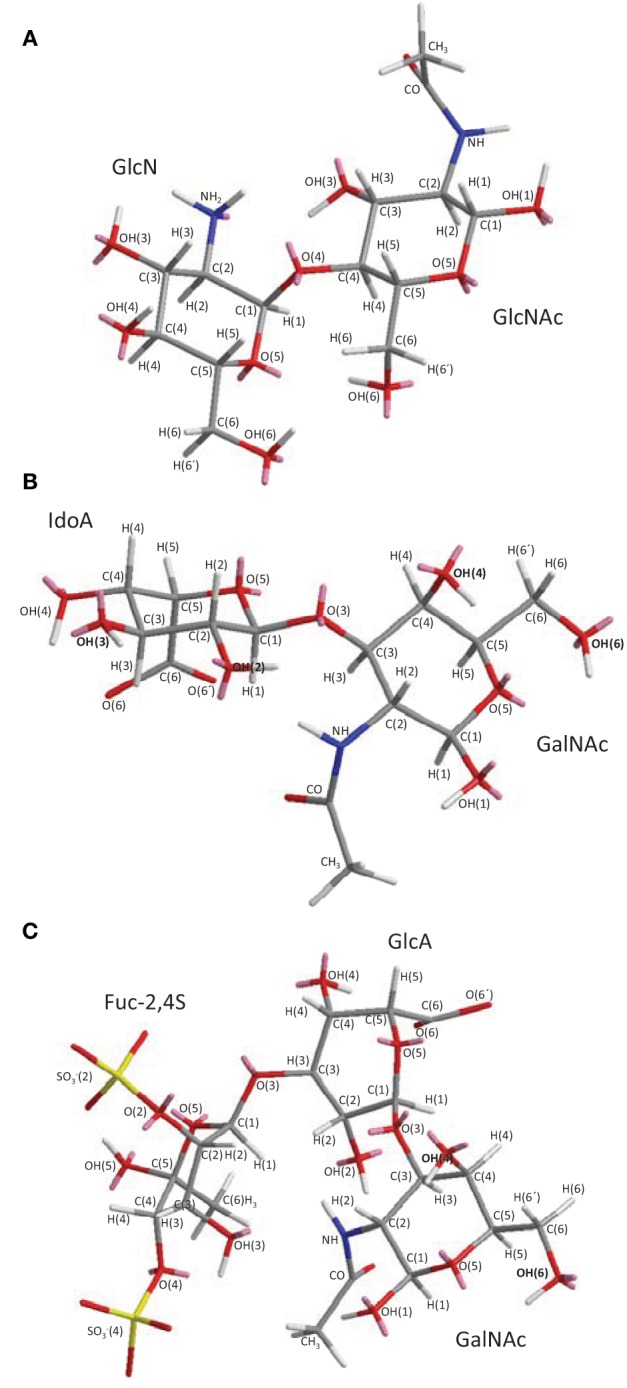
**3D structural representation of the marine glycans (A) chitin and chitosan, (B) ascidian dermatan sulfates (DSs), and **(C)** sea-cucumber fucosylated chondroitin sulfate (FucCS).** These pictures represent the lowest-energy conformations obtained by computational simulation on Chem3D Ultra 8.0 software using 10,000 step intervals of 2.0 fentosecond each, at 298 K and heating/cooling rate of 1000 Kcal/atom/ps. **(A)** Chitin and chitosan are composed of β-(1–4)-linked D-glucosamine (GlcN) and *N*-acetyl D-glucosamine (GlcNAc) units with different amounts. Chitin has ≥70% GlcNAc units while chitosan is composed of ≤30% of this same unit. **(B)** The DS from ascidian *Styela plicata, Halocynthia pyriformis*, and *Phallusia nigra* are composed of [→4)-α-L-IdoA-(2R^1^,3R^2^)-(1→3)-β-D-GalNAc-(4R^3^, 6R^4^)-(1→]_*n*_ with different sulfation patterns (Pavão et al., 1995, 1998). *S. plicata* DS has R^1^, R^2^, R^3^, and R^4^ at 66, <5, 94, 6%, respectively. *H. pyriformis* DS has R^1^, R^2^, R^3^, and R^4^ at 70, <5, 99, 1%, respectively. *P. nigra* DS has R^1^, R^2^, R^3^, and R^4^ at 80, <5, <5, and 100%, respectively. **(C)** The FucCS from *Ludwigothurea grisea* composed of {→4)-β-D-GlcA-3[→1)-α-L-Fuc*p*-2,4-di(OSO^−^_3_)]-(1→3)-β-D-GalNAc-(1→]_*n*_ (Vieira and Mourão, 1998; Vieira et al., 1991; Mourão et al., 1996; Fonseca et al., 2010). IdoA, GalNAc, GlcA, and Fuc*p* stand for iduronic acid, *N*-acetyl galactosamine, glucuronic acid, and fucopyranosyl units. Carbon (C), oxygen (O), hydrogen (H), sulfur (S) and nitrogen (N) atoms are represented in gray, red, white, yellow, and blue, and indicated by numbers within their positions in the sugar rings. The unpaired electrons of oxygens and nitrogens are shown in pink. The OH groups in **(B)** DS and **(C)** FucCS molecules that can be substituted by sulfate ester groups are highlighted in bold for rapid visualization.

Chitin is the second most abundant polysaccharide on earth after cellulose. Cellulose is mostly terrestrial while chitin is marine and terrestrial. In the marine environment, chitin is certainly the most abundant biopolymer. Chitin is structurally composed of 2-acetamino-D-glucose, also named *N*-acetyl D-glucosamine (GlcNAc), and 2-amino-D-glucose also known as D-glucosamine (GlcN) units. These units are linked by β(1 → 4) glycosidic bonds (Figure 1A). In chitin the GlcNAc content is above 70% of the total monosaccharide. This implies that this polysaccharide is highly *N*-acetylated. This in turn significantly decreases its hydrosolubility property. Low hydrosolubility levels give rise to the main natural function of chitin, which is to create a protective surface in invertebrate and fungal organisms. The major examples are exoskeletons in arthropods, especially insects and arachnids, shells in crustaceans and mollusks and cell walls in fungi.

The unique structure and particular physicochemical properties of chitin make this glycan very useful to industries of several kinds. Chitin, its derivatives, and enzymes involved in their processing are all globally explored by manufacturers of cosmetics and food products. Chitin is also used by agricultural, pharmaceutical, and biomedical companies. However, the interest and application in medicine clearly surpasses any other area (Sugano et al., 1980; Suzuki et al., 1982; Nishimura et al., 1986; Bourbouze et al., 1991; Fukada et al., 1991; Ikeda et al., 1993; Maezaki et al., 1993; Deuchi et al., 1995; Bleau et al., 1999; Shibata et al., 1997, 2000; Cho et al., 1998; Khor, 2001; Barone et al., 2003; Okamoto et al., 2003; Qian and Glanville, 2005; Di Rosa et al., 2005; Malaguarnera et al., 2005; Owens et al., 2006; Zhou et al., 2006; Harish Prashanth and Tharanathan, 2007; Jayakumar et al., 2007; Bonferoni et al., 2008; Liu et al., 2008; Wu et al., 2008; Yang et al., 2008; Muzzarelli, 2009; Paolicelli et al., 2009; Perioli et al., 2009; Tan et al., 2009).

The structure of chitin polymers can be found at three forms, α, β, and γ. The α-chitin is known to have a parallel-sheet conformation and is the most abundant form in nature. This form can be found in the shells of crabs and shrimps. The β-chitin is found in the spines of diatoms, squid pens, and pogonophoran tubes. The β-chitin polymers are made of anti-parallel sheets. The γ-chitin, which occurs in fungi and yeast, is comprised of both α and β forms, thus, having a mixture of both anti-parallel and parallel sheets.

Chitin, which has a compact conformation made of highly acetylated regions and sheet-rich 3D-structures, is poorly water-soluble. These properties make industrial and commercial exploration of this structure difficult. To enhance hydrosolubility, chemically modified or hydrolyzed derivatives are usually generated. For example, alkaline hydrolysis removes the acetyl groups and leaves just the amino groups allowing the polymer to be converted from a poorly water-soluble molecule into a highly water-soluble one.

Chitosan is a cationic polysaccharide made up of the same units and glycosidic linkage of chitin (Figure 1A). However, low amounts of GlcNAc are found in chitosan, usually less than 30%. Physicochemical characteristics like hydrophobicity and inter-chain interactions depend on the amount and distribution of acetyl groups. Another physicochemical characteristic that varies naturally among different chitosan samples is the molecular weight (MW). Based on this characteristic, three categories of chitosan exist. These categories are named accordingly to their different MWs: high molecular weight chitosans (HMWC), medium molecular weight chitosans (MMWC), and low molecular weight chitosans (LMWC). The MW ranges between 10–100 kDa for LMWC, 100–300 kDa for MMWC, and over 300 kDa for HMWC. In aqueous solution the HMWC sample are more viscous than those prepared with LMWC or MMWC polymers. Although LMWC can be obtained by size exclusion chromatography of unmodified chitosans, enzymatic methods can be additionally employed to produce LMWC derivatives. Although chitosan and its derivatives are all cationic by nature, structural differences among them account for differences in their biological activities and physicochemical properties (Zhang et al., 2009; Ozhan Aytekin et al., 2012).

Chitin and chitosan are widely explored as dietary supplements. Some pharmaceutical functions of chitin and chitosan occur due to their unique physicochemical properties as naturally occurring molecules. They are non-toxic, renewable, and biodegradable. Depending on structures, they exert antitumor, immunoenhancement, antimicrobial, and hypocholesterolemic properties. These properties and activities make these polymers very promising therapeutic candidates (Ilium, 1998). Other therapeutic applications of chitin and chitosan are also under current investigation. Examples are their multiple effects in drug delivery and gene therapy. These activities include ocular, nasal, and vaginal delivery as well as targeted delivery into tumor sites, colon, and wound dressing (bandages). These two marine carbohydrates have also the capability in interacting with receptors on macrophage surfaces to stimulate an immune response in cells (Muzzarelli, 2009) as detailed further. Other clinical effects are also discussed below.

### Effects on immune response

It has been shown that chitin microparticles are effective in clinical treatments including tumor cases, bacterial and viral infections (Suzuki et al., 1982; Nishimura et al., 1986; Shibata et al., 1997). Administration of these particles through the vascular system enhances the generation and release of cytokines by macrophages. The action of chemokines responsible to activate leukocytes in immunological events is mediated by various surface receptors. These receptors act as agents that help internalization of chitin microparticles. Because of the stimulatory action on macrophages, it is believed that chitin plays a pivotal role in depressing allergen-induced type 2 inflammatory responses. This belief is supported by the fact that cytokines are involved in the regulation of allergic immune responses (Shibata et al., 2000). Moreover, it is also known that chitin is a T helper cell type 1 (Th1) adjuvant agent. It has the ability to up-regulate Th1 immunity at the same time it down-regulates T helper cell type 2 (Th2) immunity. The principal type of chitin with this property is the shrimp α-chitin. Microparticles made of shrimp α-chitin have the ability to convert an allergic response mediated by Th2 immunity into an inflammatory response mediated by Th1 immunity (Muzzarelli, 2009).

Studies in mammals have shown that in cases of infection, chitinase enzymes can increase immunity (Bleau et al., 1999). This finding was supported by trials on allergic and asthmatic patients in which macrophages have shown increased expression of acidic mammalian chitinase (AMCase) (Barone et al., 2003; Di Rosa et al., 2005; Malaguarnera et al., 2005). Although some researches of chitin-related enzymes has clearly pointed toward beneficial properties in immunologic system, the specific roles of such enzymes in host defense mechanisms as possible therapeutic agents are yet to be uncovered.

### In formulations for drug delivery

In the recent years of the glycomics age, researches about drug delivery and development has placed a great deal on chitosan due to its capacity of addressing drugs to target tissues. This can be done efficiently by different administration routes such as nasal, oral, intra-peritoneal, and intravenous. Some outcomes provided by these different routes of administration or targeted treatments using chitosan molecules are shown in Table 1.

**Table 1:** **Successful applications of chitin and chitosan in drug delivery**.

**Delivery systems**	**Application**	**References**
Ocular delivery	Ocular nanomedicines to be used in clinical practices from chitosan-based nanosystems	Zhang et al., 2009
Nasal delivery	Insulin transportation due to mucoadhesive, cationic and biodegradable properties of PEG-g-chitosan nanoparticles	Paolicelli et al., 2009
Targeted delivery to tumors	Reduction of systematic cytotoxicity, inhibition of cancer cell growth, induction of apoptosis of bladder tumor cells	Tan et al., 2009
Vaginal delivery	Mucoadhesion, enhanced penetration, peptidase inhibition by chitosan containing tablets	Perioli et al., 2009
Wound dressing	Healing of wounded soft tissue, bone, nerve, cartilage by chitin and chitosan based materials	Bonferoni et al., 2008

### Hypocholesterolemic and hypolipidemic properties

As hypocholesterolemic and hypolipidemic agents, chitosan molecules can lower the total cholesterol, plasma and liver triacylglycerol levels quite effectively (Sugano et al., 1980; Fukada et al., 1991; Ikeda et al., 1993; Maezaki et al., 1993; Cho et al., 1998). These activities have been reported with little or no drastic side effects. Chitosans of different MW exhibit distinct effects (Maezaki et al., 1993). The varying activity was demonstrated by *in vitro* studies using LMWC derivatives of different MW ranges. Results have indicated that LMWC derivatives of different MWs have different fat-binding and bile-salt-binding capacities (Zhou et al., 2006; Liu et al., 2008). Another influencing factor in binding properties of chitosan fibers is the particle size of LMWC derivatives. Powdered forms of chitosan have shown to have higher binding capacities when compared to flake forms. The hypocholesterolemic activity of LMWC derivatives may be explained by electrostatic attraction and absorption mechanisms with bile-salts and fatty acids. In the stomach, LMWC derivatives entrap fat droplets when chitosan fibers and fat are consumed together. This entrapment mechanism leads to precipitation of the fat molecules together with LMWC derivatives, which leads to formation of clusters at neutral pH in the small intestine. This prevents fat digestion (Deuchi et al., 1995; Zhou et al., 2006). This is a procedure widely explored by pharmaceutical industries to develop dietary and health care chitosan-based products, mainly used for weight control or reduction. Nevertheless, the ability to reduce fat-absorption by LMWC fibers is likely to be significantly lower or nonexistent if very acidic conditions are found in the stomach.

### Effects on hemostasis

Pure chitin/chitosan fibers have wound healing and blood coagulating properties. They can be used either as internal hemostatic dressing or as hemostatic bandages (Qian and Glanville, 2005; Harish Prashanth and Tharanathan, 2007; Jayakumar et al., 2007; Khor, 2001). Purity levels of this marine glycan are influential for these activities. This molecule is mostly obtained from shells of marine organisms and, during isolation procedures, other naturally occurring molecules can be co-extracted as contaminants. Studies have demonstrated that depending on the dose and purity, both chitin and chitosan are significantly effective on decreasing the blood coagulation time (BCT) (Okamoto et al., 2003). In this work, the effects of both chitin and chitosan on blood coagulation and platelet aggregation (PA) were evaluated using canine blood in *in vitro* experiments. Whole blood was mixed with chitin and chitosan suspensions (0.0001–1.0 mg/ml), and then the BCT was measured. Chitin and chitosan have been proven to reduce BCT in a dose-dependent manner. Platelet-rich plasma (PRP) was mixed with chitin- and chitosan-suspensions, and then PA was measured in a dual aggregometer. The PA level induced by chitin was the strongest of all samples tested including chitosan, cellulose and latex used as comparative standards. When washed platelets were used, the PA level induced by chitin was similar to that of chitosan, while the rate of coagulation was lower than that of PRP. Chitin and chitosan have shown the ability to enhance the release of platelet derived growth factor-AB (PDGF-AB) and transforming growth factor-β (TGF-β) from platelets (Okamoto et al., 2003).

The hemostatic effect of chitosan as an internal dressing agent against bleeding of liver, aorta, lung, kidney, and cardiac ventricle wounds have been tested and certified by *in vivo* experiments (Owens et al., 2006). Hemostatic property of chitosan may benefit patients with coagulopathies since this therapeutic property is independent of coagulation (co)factors (Yang et al., 2008; Zhang et al., 2009). The beneficial activity of chitosan depends almost entirely on platelets, as supported previously (Okamoto et al., 2003; Wu et al., 2008). *In vitro* experiments have proven that the hemostatic activity of chitosan can contribute effectively to PA and adhesion (Zhang et al., 2009). Therefore, serpin-dependent and -independent anticoagulant and antithrombotic pathways are not involved in the effect of chitosan.

### Effects against cancer

Enzymes that are involved in chitin/chitosan synthesis and degradation are generally named glycosyltransferases and glycosidases, respectively. They are highly specific in terms of reaction. In biosyntheses, for instance, the presence and amounts of the correct substrate, sugar donors, and enzyme dictate whether the reaction will occur or not. These enzymes have been noted to be expressed in different levels accordingly to healthy or pathological conditions. The over- or down-expression of these enzymes will result in significant changes of the structures of the cellular glycans. Therefore, the structural integrity of the surface glycans at the surface of healthy cells is intimately controlled by the activities of glycosyltransferases and glycosidades. A small change in the balance of the activities of these two enzymes can lead to diseases (Ohtsubo and Marth, 2006). Studies have demonstrated that changed expressions of these enzymes are in fact indicators of carcinogenesis. For example, the β(1 → 6) branch levels of *N*-linked glycans, found between mannose (Man) and GlcNAc units are seen to be increased in tumor cases. Interestingly, these units are products from digestions of chitin and chitosan polysaccharides. More specifically, the structure GlcNAc-β(1 → 6)-Man-α(1 → 6)Man-β results from a combination of available substrate (the digested chitin/chitosan) and the specific glycosyltransferase, N-acetylglucosaminyltransferase-V (GnT-V) (Humphries et al., 1986; Fernandes et al., 1991; Handerson and Pawelek, 2003; Dube and Bertozzi, 2005; Wattenberg, 2006). *In vivo* studies have shown that β(1 → 6) GlcNAc branching, catalyzed by GnT-V activity, is intimately related with carcinogenesis (Wattenberg, 2006). In terms of therapy, the regulation of the up-take levels of chitin and chitosan and the control of the enzyme activities related with the degradation of these polymers, by gene and/or enzymatic therapy, are effective clinical routes to decrease availability of substrates used to build up glycans involved in tumor development.

In addition to what has been mentioned above, chitin synthase and chitinase that work on synthesis and degradation of chitin, respectively, have also shown to play a key role in invasion by many pathogens, including tumor cells. Hence, inhibitors of chitin synthases might have therapeutic uses in cancer. In fact, several reports using *in vitro* and *in vivo* experiments have pointed out that plant and bacterial chitinases are indeed effective agents in cancer regressions (Pan et al., 2005; Sotgiu et al., 2008; Xu et al., 2008).

## Sulfated polysaccharides

Marine GAGs have different structures than those present in common mammal GAGs. For example, dermatan sulfate (DS) isolated from the ascidian species *Phallusia nigra* is composed of [→4)-α-L-IdoA-(2R^1^,3R^2^)-(1→3)-β-D-GalNAc-(4R^3^,6R^4^)-(1→]_*n*_, where IdoA is iduronic acid, GalNAc is *N-acetyl* galactosamine, R^1^, R^2^, R^3^, and R^4^ are sulfate groups at 80, <5, <5, and 100 percent, respectively, (Figure 1B) (Pavão et al., 1995). Conversely, the commonest mammalian DS is mostly composed of 2-sulfated IdoA units together with occasional C4 sulfation at GalNAc units. Another different GAG from marine invertebrates is fucosylated chondroitin sulfate (FucCS) isolated from the sea-cucumber *Ludwigothurea grisea*, which is composed of [→4)-β-D-GlcA-3[→1)-α-L-Fuc*p*-2,4-di(OSO^−^_3_)]-(1→3)-β-D-GalNAc-(1→]_*n*_, in which GlcA is glucuronic acid, and Fuc*p* is a fucopyranosyl residue (Figure 1C) (Vieira and Mourão, 1998). Conversely, the commonest chondroitin sulfate (CS) in mammals is composed of [→4)-β-D-GlcA-(1→3)-β-D-GalNAc-(1→]_*n*_ where its GalNAc units can be either mostly 4-sulfated (CS-A) or predominantly 6-sulfated (CS-C) (Pomin et al., 2012).

**Figure 2:**
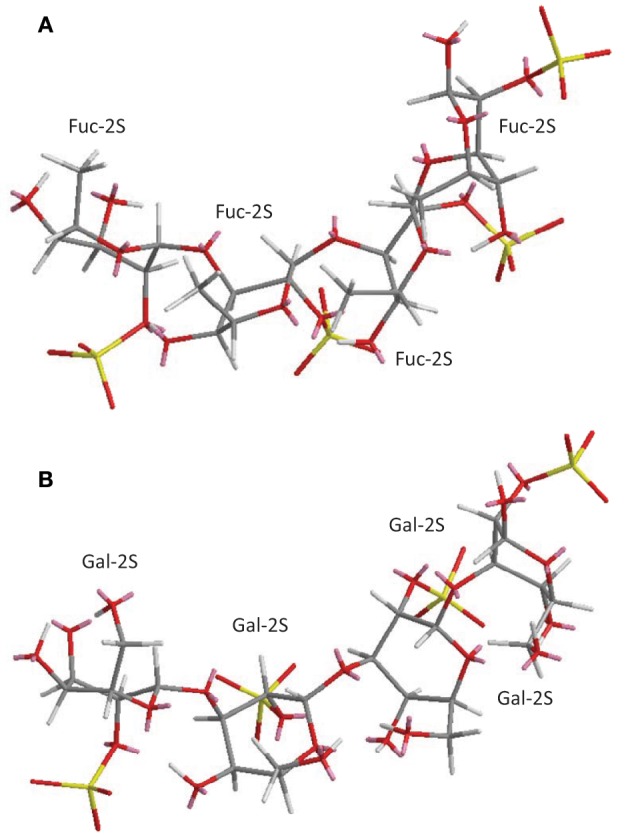
**3D structural representation of the sea-urchin 3-linked 2-sulfated glycans: (A) sulfated fucan (SF) from *Strongylocentrotus franciscanus*, and **(B)** sulfated galactan (SG) from *Echinometra lucunter* both shown at their tetrasaccharide models.** These pictures represent the lowest-energy conformations obtained by computational simulation on Chem3D Ultra 8.0 software using 10,000 step intervals of 2.0 fentosecond each, at 298 K and heating/cooling rate of 1000 Kcal/atom/ps. The polymers are made of the following structures **(A)** [→3)-α-L-Fuc*p*-2(OSO^−^_3_)-(1→]_n_ (Alves et al., 1997) for sea-urchin *S. franciscanus*, and **(B)** [3)-α-L-Gal*p*-2(OSO^−^_3_)-(1→]_n_ (Vilela-Silva et al., 1999) for sea-urchin *E. lucunter*. Gal*p* and Fuc*p* stand for galactopyranosyl and fucopyranosyl units, respectively. Carbon (C), oxygen (O), hydrogen (H), and sulfur (S) atoms are represented in gray, red, white, and yellow. They have not been indicated because of the big conformational overlap. The unpaired electrons of oxygens are shown in pink.

As opposed to ascidian DS and sea-cucumber FucCS which are heterogeneous marine sulfated polysaccharides (MSPs) in terms of monosaccharide composition, the SFs and SGs are very homogeneous given that they are composed of only Fuc*p* or galactopyranose (Gal*p*) units distributed in a quite regular and repeating backbone (Table 2). The major differences between species from SFs or SGs are either the sulfation pattern or the glycosidic linkage type (Figure 2 and Table 2). From comparative studies using the SFs and SGs shown in Table 2, their biomedical responses can be understood based on some structural requirements (Pereira et al., 2002). This analytical procedure helps to uncover the underlying mechanisms of action of their biomedical effects through a very accurate and efficient way. Some of the results in these advanced structure-function relationship studies are detailed below.

**Table 2:** **Oligosaccharide repetitive units of SFs and SGs from echinoderms sea-urchins (Echinoidea), and sea-cucumber (Holothuroidea), red algae (Rhodophyta), and ascidians or tunicates (Ascidiacea)**.

**Species (class)**	**Structure**
*Ludwigothuria grisea* (holothurioidea)	[→3)-α-L-Fucp-2,4(OSO^−^_3_)-(1→3)-α-L-Fuc*p*-(1→3)-α-L-Fuc*p*-2(OSO^−^_3_)-(1→3)-α-L-Fuc*p*-2(OSO^−^_3_)-(1→]_n_
*Strongylocentrotus purpuratus* II (echinoidea)	[→3)-α-L-Fuc*p*-2,4di(OSO^−^_3_)-(1→3)-α-L-Fuc*p*-4(OSO^−^_3_)-(1→3)-α-L-Fuc*p*-4(OSO^−^_3_)-(1→]_n_
*Strongylocentrotus purpuratus* I (echinoidea)	80% [→3)-α-L-Fuc*p*-2,4di(OSO^−^_3_)-(1→]_n_ and 20% [→3)-α-L-Fuc*p*-2(OSO^−^_3_)-(1→]_n_
*Strongylocentrotus franciscanus* (echinoidea)	[3)-α-L-Fuc*p*-2(OSO^−^_3_)-(1→]_n_
*Strongylocentrotus droebachiensis* (echinoidea)	[→4)-α-L-Fuc*p*-2(OSO^−^_3_)-(1→]_n_
*Strongylocentrotus pallidus* (echinoidea)	[→3)-α-L-Fuc*p*-2(OSO^−^_3_)-(1→3)-α-L-Fuc*p*-2(OSO^−^_3_)-(1→3)-α-L-Fuc*p*-4(OSO^−^_3_)-(1→3)-α-L-Fuc*p*-4(OSO^−^_3_)-(1→]_n_
*Lytechinus variegatus* (echinoidea)	[→3)-α-L-Fuc*p*-2(OSO^−^_3_)-(1→3)-α-L-Fuc*p*-2(OSO^−^_3_)-(1→3)-α-L-Fuc*p*-4(OSO^−^_3_)-(1→3)-α-L-Fuc*p*-2,4di(OSO^−^_3_)-(1→]_n_
*Arbacia lixula* (echinoidea)	[→4)-α-L-Fuc*p*-2(OSO^−^_3_)-(1→4)-α-L-Fuc*p*-2(OSO^−^_3_)-(1→4)-α-L-Fuc*p*-(1→4)-α-L-Fuc*p*-(1→]_n_
*Echinometra lucunter* (echinoidea)	[→3)-α-L-Gal*p*-2(OSO^−^_3_)-(1→]_n_
*Glyptosidaris crenularis* (echinoidea)	[→3)-β-D-Gal*p*-2(OSO^−^_3_)-(1→3)-β-D-Gal*p*-(1→]_n_
*Botryocladia occidentalis* (rodophyta)	[→3)-β-D-Gal*p*-2R_1_-3R_2_-(1→4)-α-D-Gal*p*-2R_3_-3R_4_-(1→]_n_, where R_#_ = OSO^−^_3_ or OH, R_1_ and R_2_ = OSO^−^_3_ in ~66 and 33%, respectively
*Gelidium crinale* (rodophyta)	[→3)-β-D-Gal*p*-2R_1_-4R_2_-(1→4)-α-D-Gal*p*-2R_3_-3R_4_-(1→]_n_, where R_#_ = OSO^−^_3_ or OH, R_1_ and R_2_ = OSO^−^_3_ in ~60 and 15%, respectively
*Styela plicata* (ascidiacea)	{→4)-α-L-Gal*p*-2[→1)-α-L-Gal*p*]-3(OSO^−^_3_)-(1→}_n_
*Hedmania monus* (ascidiacea)	[→4)-α-L-Gal*p*-3(OSO^−^_3_)-(1→]_n_

Besides the unique structures of the MSPs, they also show differential medical properties (Cumashi et al., 2007). This is especially evident when compared to the common mammalian SPs, GAGs. The medical properties of MSPs are directly related to some of their unique structural features, which are not found in mammalian counterparts. For example, while the mammalian CS, which lacks a fucosyl branch, is a non-anticoagulant polysaccharide, the marine FucCS is anticoagulant since it naturally bears the fucosyl branch (Mourão et al., 1996). If this branch is removed in the MSP, for example, by mild acid hydrolysis, it becomes inactive as anticoagulant (Mourão et al., 1996). Below, some of these unique structural requirements necessary to achieve a good response in the medical actions of the MSPs will be described. This will be made through a systematic discussion about the structure-function relationship in the medical activities of the ascidian DS, sea-cucumber FucCS, sea-urchin and red algal SFs and SGs whose mechanisms of action have been elucidated. The events in which these mechanisms of action have been elucidated are inflammation, coagulation, thrombosis, cancer, and angiogenesis.

### Anti-inflammatory effects

**Figure 3:**
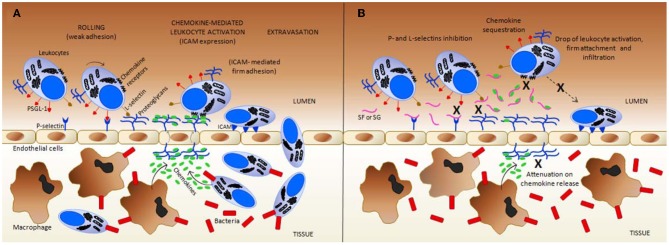
**Simplified scheme regarding the inflammation mechanisms in (A) normal (untreated) vs. **(B)** the treated condition with exogenous sulfated fucans (SFs) and sulfated galactans (SGs).** These glycans can target multiple points during the inflammatory process. **(A)** In response to an inflammatory stimuli, such as a bacterial infection, resident macrophages in inner tissues produce both chemokines that attract more leukocytes into these inflamed tissues, and cytokines (such as tumor necrosis factor, TNF) that trigger, at the early stages, the display of pre-formed P-selectins on the luminal surface of endothelial cells (the cytokine-induced P-selectin exposure is not shown at the panels). Cytokines can also induce the expression of E-selectin by endothelial cells (mechanism not shown). GAGs at endothelial proteoglycans play an important role in L-selectin binding, in chemokine presentation to chemokine receptors on neutrophils, and in the transportation of chemokines produced by tissue macrophages and further infiltrated leukocytes. Intercellular adhesion molecule (ICAM), and P-selectin glycoprotein ligand-1 (PSGL) are important leukocyte cell-membrane proteins involved in rolling and firm adhesion, respectively. **(B)** In the presence of SFs, and likely SGs, by direct contact, both P- and L-selectins are blocked to interact further with PSGL-1, and GAGs, respectively, thus, causing a reduction on the leukocyte recruitment. In addition, at certain concentrations, SFs and SGs sequestrate the chemokines responsible to drive and to activate the leukocytes. This is another anti-inflammatory action of these marine glycans. This sequestration occurs most likely because of the presence of conserved heparin-binding sites (BBXB motifs, where B and X are basic and neutral amino acids) in some pro-inflammatory chemokines such as CCL5/RANTES. Due to chemokine sequestration, the numbers of activated defense cells, their firm attachment to the endothelial surface and further infiltration become all consequently reduced in treatment cases. Besides those actions, the number of released chemokine as a pro-inflammatory feedback process from inner tissues is also attenuated due to the decreased number of infiltrated cells. This latter event enhances the anti-inflammatory activity of the MSPs. All mechanisms marked by X in **(B)** collaborate in conjunction to the resultant anti-inflammatory action of SFs and SGs. Figure reproduced with permission from (Pomin, 2012b).

When some structural requirements are present, the MSPs (ascidian DS, sea-cucumber FucCS and sea-urchin or algal SFs and SGs) may exhibit anti-inflammatory activities, as observed by *in vitro* and *in vivo* experiments (Borsig et al., 2007; Cumashi et al., 2007; Melo-Filho et al., 2010; Belmiro et al., 2011; Kozlowski et al., 2011; Pomin, 2012b,c). The anti-inflammatory action of these MSPs essentially resides in abrogating the P- and L-selectin-mediated leukocyte trafficking, and recruitment and the chemokine-related leukocyte activation during inflammatory events. Hypotheses that the MSPs can also sequester chemokines also exist (Pomin, 2012b). Hence, the MSPs may exhibit anti-inflammatory activities via both cellular and molecular mechanisms of inflammation. A detailed description of the mechanisms of action is illustrated in Figure 3 for SFs and SGs used as examples. It seems that the same mechanisms of action also occur for the ascidian DS and the sea-cucumber FucCS (Borsig et al., 2007; Melo-Filho et al., 2010; Belmiro et al., 2011; Kozlowski et al., 2011).

As seen in most steroidal anti-inflammatory drugs, such as the glucocorticoids, downside immunosuppressive effects for the above-mentioned anti-inflammatory mechanisms of the MSPs can exist. Since the extravasation of leukocytes to the sites of infection are impaired by the use of MSPs in optimal anti-inflammatory doses, the lower levels of leukocytes at the infected or injured sites are somewhat disrupted. This can decrease the ability of patients to fight infections.

The work of Melo-Filho and coworkers has shown that the sea-cucumber FucCS can greatly attenuate progression of renal fibrosis. This was observed using animals submitted to unilateral ureteral obstruction. The anti-fibrotic mechanism occurs through the stoppage of the P-selectin-driven cell migrations (Melo-Filho et al., 2010). In this work essentially based on *in vivo* experiments, mice were given 4 mg/kg body weight of FucCS intraperitoneally, once a day. After 14 days of injection, their kidneys were examined by histological, immune-histochemical, and biochemical methods. Compared with control mice, collagen deposition decreased in the course of renal fibrosis in the mice receiving FucCS as revealed by Sirius red staining and hydroxyproline content. The cellularity related to myofibroblasts and macrophages was also clearly reduced, as was the production of TGF-β. Fibrosis induced by unilateral ureteral obstruction was observed markedly decreased in P-selectin-deficient mice, which was also proved insensitive to the invertebrate GAG. In this reference, the authors have clearly demonstrated the attenuation ability of FucCS in renal fibrosis using the ureteral obstruction model in mice. As conclusion, the anti-inflammatory mechanism in which FucCS works is mostly driven by P-selectin-mediated cell migration (Melo-Filho et al., 2010).

The phenomenon of P-selection blocking activity by FucCS was demonstrated again in the work of Borsig and co-authors (Borsig et al., 2007). In this work, the authors have shown by *in vitro* experiments that not only heparin can block P- and L-selection, but also the sea-cucumber FucCS. The blocking action of these GAGs impairs the binding of selectins with sialyl Lewis(x). This blocking action disrupts the rolling and migration of the leukocytes on the vessel surfaces close to the inflamed sites. The sea-cucumber FucCS was proven to be a potent inhibitor of P- and L-selectin binding to immobilized sialyl Lewis(x), and of LS180 carcinoma cell attachment to immobilized P- and L-selectins. Inhibitions have been shown to occur in a concentration-dependent manner. Interestingly, FucCS was 4–8-fold more potent than heparin in the inhibition of P- and L-selectin-sialyl Lewis(x) interactions. No inhibition of E-selectin was observed. This was expected based on similar studies undertaken by Cumashi and coworkers on the anti-inflammatory activity of some brown algal SFs (Cumashi et al., 2007). In the work of Borsig et al. (2007), FucCS demonstrated to have inhibitory properties on lung colonization of adenocarcinoma MC-38 cells in an experimental metastasis using mice. This inhibitory activity was also observed in neutrophil recruitment in two *in vivo* models of inflammation (thioglycollate-induced peritonitis and lipopolysaccharide-induced lung inflammation). Inhibition occurred at a dose that produces no significant change in plasma activated partial thromboplastin time (aPTT). Removal of the sulfated fucose branches in the FucCS (Figure 1C) abolished its inhibitory effect as observed by both *in vitro* and *in vivo* experiments. This proves the importance for the fucosyl branch for this activity. The results from this reference suggest that invertebrate FucCS may be a potential alternative to heparin for blocking metastasis and inflammation without the undesirable anticoagulant side effects seen in heparin.

Another beneficial aspect of MSPs was shown in studies of the anti-inflammatory potential of ascidian DS with different structures (Figure 1B) (Belmiro et al., 2011; Kozlowski et al., 2011). Subcutaneous administration of ascidian DS has shown therapeutic effects against colon inflammation in rats by reducing macrophage and T-cell recruitment and activation. These activities are in perfect coherence with the mechanisms described in Figure 3. The work of Belmiro also showed the capacity of DS as an anti-inflammatory agent in decreasing the myofibroblast population in fibrosis-induced mice submitted to unilateral ureteral obstruction. The *in vivo* experiment used was similar to that used in the work of Melo-Filho et al. (2010). In the work of Kozlowski, the investigators showed *in vivo* anti-inflammatory action of two ascidian DSs. The conclusion was based on the ascidian DS capacity to block infiltration of defense cells in a thioglycollate-induced peritonitis mouse experiment (Kozlowski et al., 2011).

Cumashi and coworkers have shown anti-inflammatory effects of some brown algal SFs using *in vitro* assays to test the binding properties of the MSPs with selectins. Curiously, the brown algal heterogenous SFs (also known as fucoidans) were able to clear inhibit P- and L-selectins but not E-selectin (Cumashi et al., 2007).

### Anticoagulation and antithrombosis: the serpin-independent mechanism

**Figure 4:**
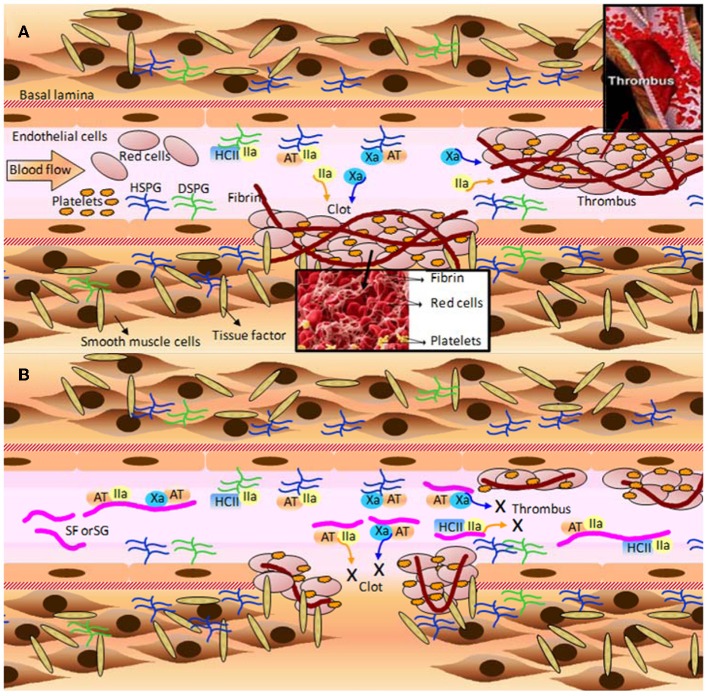
**A few of the molecular and cellular players in (A) blood coagulation, clot and thrombus formation; and **(B)** the anticoagulant and antithrombotic mechanisms of the marine sulfated fucans (SFs) and sulfated galactans (SGs). (A)** When the blood vessel wall is disrupted by an injury (atherosclerotic plaque or a physical rupture, for example) tissue factors normally expressed and localized below the basal lamina become exposed to the blood stream. Blood factor XII is recognized by tissue factor, and after making complex with it, becomes factor XII activated (XIIa) (complex not shown). XIIa initiates the blood coagulation cascade leading to the expressive formation of thrombin (IIa) and factor X activated (Xa). These factors feed the formation of more blood coagulation factors which will result in clot or thrombus formation. Thrombin acts directly on fibrinogen in order to form fibrin fibers, which stabilizes the clots and thrombus through cross-linked fibers. Platelets play an important role to this stabilization as well. The natural inhibitors of the two proteases (Xa and IIa) are the serpins antithrombin (AT), and heparin cofactor II (HCII). AT is able to act directly on either Xa or IIa, whereas HCII acts only on IIa. Upon interaction with heparan sulfates and dermatan sulfates of proteoglycans distributed throughout the endothelial surface of blood vessels, AT and HCII become activated for inhibiting actions. This leads to sequestration of the plasma soluble Xa and IIa factors. It is worth to mention that AT is a heparin-binding protein with the BBXB motif of high-affinity to SPs. HSPG and DSPG stand for heparan sulfate and dermatan sulfate proteoglycans, respectively. **(B)** The inhibitory mechanisms provoked by MSPs are analogous to the natural inhibitory mechanisms caused by the proteoglycans at surfaces of the vessels. However, due to the large plasmatic amounts of SFs and SGs in treatment conditions, the cofactors AT and HCII would have their natural inhibitory actions enhanced by certain orders of magnitude, consequently lowering the plasmatic concentration of active factors IIa and Xa. The decreased amounts of these blood factors abrogate the clotting and thrombus formation, as a consequent result. Fibrinolytic activity is responsible to undertake metabolic process on formed clots and thrombus after significant inactivation of the proteases Xa and IIa. All the mechanisms marked by X in **(B)** lead to the anticoagulant and antithrombotic actions of SFs and SGs. Figure reproduced with permission from (Pomin, 2012b).

The effects of MSPs on hemostasis are the mostly studied medical activities of these compounds. A detailed scheme describing their major mechanism of action, as possible anticoagulants and antithrombotics, is provided at Figure 4, in which SFs and SGs are used as examples. The mechanisms of action reside on the inhibition of some coagulation proteases like thrombin (IIa) and factor Xa, via their physiological inhibitors, named serpins (serine-protease inhibitors). The most common serpins of this system are antithrombin (AT) and heparin cofactor II (HCII). Although at different degrees of response, the majority of the MSPs described herein: the ascidian DS (Figure 1B) (Vicente et al., 2004; Kozlowski et al., 2011), the sea-cucumber FucCS (Figure 1C) (Mourão et al., 1996; Mourão, 2004), the algal SFs and SGs (Table 2) (Pereira et al., 1999; Farias et al., 2000; Mourão, 2004; Pomin and Mourão, 2012) and the invertebrate SFs or SGs (Figure 2 and Table 2) (Pereira et al., 1999; Farias et al., 2000; Pomin, 2012b), have all effects in this serpin-dependent mechanism (Figure 4). The anticoagulant effects of the MSPs are intimately dependent on some of their structural features. For example, the SF from *Strongylocentrotus franciscanus* (Figure 2A and Table 2) is not an anticoagulant polysaccharide while the SG from *Echinometra lucunter* (Figure 2B and Table 2) is anticoagulant (Pereira et al., 2002). The only difference between these two compounds is the monosaccharide type. The other features C3-glycosydic linkage, 2-sulfation, L-enatiomericity, and α-anomericity are equal (Figure 2). This single structural difference is enough to make either an active or an inactive compound.

Besides the common serpin-dependent anticoagulant activity of the FucCS from the sea-cucumber *L. grisea* (Figure 1C), and the SG from the red alga *Botryocaldia occidentalis* (Table 2), these glycans have also shown serpin-independent anticoagulant actions (Glauser et al., 2008, 2009). Initially, their anticoagulant actions were essentially attributed by their capacity in potentiate factors Xa and IIa inhibition via AT and HCII, as summarized in Figure 4. Currently, the sea-cucumber FucCS and the red algal SG are also known to inhibit the generation of factor Xa and IIa by interfering in the formation of the blood cofactor complexes at the surface of the cells. Factor Xa is activated mainly by the intrinsic tenase complex, while IIa is converted from II by the prothrombinase complex. FucCS and SG were shown the ability to inhibit the activation of these tenase and prothrombinase complexes (Glauser et al., 2008, 2009). The formation of these complexes is a key step for the generation and amplification of the coagulation cofactors. Besides this serpin-independent mechanism, another novel mechanism is the inhibition of thrombosis by targeting tissue factor in combination with factor Xa, as reported by the recent work of Zhao and coworkers using the sea-cucumber FucCS (Zhao et al., 2013).

### Therapeutic effects against cancer growth and metastasis

**Figure 5:**
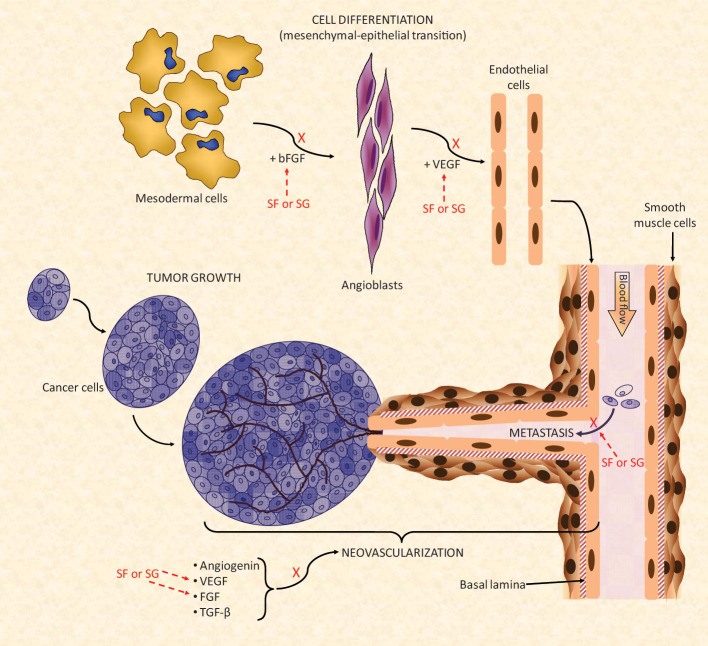
**A simplified scheme of the major biochemical mechanisms involved in tumor angiogenesis.** Multiple points of action are targeted by the SFs and SGs. For a new blood vessel to be formed and to grow properly there should be a feeding of stimulatory angiogenic factors such as angiogenin, VEGF, FGF, and TGF-β for formation of the new vessels. The mesenchymal–epithelial transition must also occur concomitantly to provide newly formed endothelial cell to help the construction of the new blood ducts. In this event, modulated also by FGF molecules, mesodermal cells undergo transition until angioblasts which is the precursors of mature endothelial cells. Under the influence of VEGF, newly formed endothelial cells will be used for building the novel vessels (Lamalice et al., 2007). Neovascularization is a fundamental process for cancer growth in primary tumors as well as to feed the tumor growth at new metastatic spots. SFs and SGs can inhibit the action of FGF and VEGF molecules either at the endothelial cell differentiation as well as during the feeding of the angiogenesis development. Interactions of SFs and SGs with these factors as well as with their respective receptors have been observed. Besides this neovascularization inhibitory function, SFs and SGs were also reported to synergically reduced tumor spreading by decreasing their cell-adhesion capacity (Croci et al., 2001) during the tumor proliferation stage. All the mechanisms marked by X collaborate in conjunction to the anti-angiogenic and/or antitumoral effects of SFs and SGs. Figure reproduced with permission from (Pomin, 2012b).

The effects of MSPs against cancer growth seem to be related to the blocking of tumor angiogenesis that feeds the growth of tumor cells (Pomin, 2012b), as illustrated in Figure 5. Like some mammal GAGs, such as heparin, MSPs have shown the capacity to bind growth factors such as basic fibroblast growth factor (bFGF) and vascular endothelial growth factor (VEGF). This binding will impair, respectively, the differentiation of mesodermal cells into angioblasts and angioblasts into endothelial cells (Figure 5). These cellular differentiations are important to the neovascularization process (Figure 5). Several articles have demonstrated the capacity of MSPs in binding with these growth factors (Tapon-Bretaudière et al., 2000, 2002; Cumashi et al., 2007).

Besides interfering in tumor neovascularization, the MSPs have also the capacity to inhibit, to some extent, the metastasis of tumor cells. This action is driven by blocking the adhesion capacity of the tumor cell onto the surface of the blood vessels (Figure 5) (Croci et al., 2001; Borsig et al., 2007; Kozlowski et al., 2011). This step is crucial for proper migration and invasion of the primary and mature cancer cells toward new spots of growth (metastasis). The mechanism of action of this tumor adhesion inhibition by MSPs seems to be related to the blocking of P- and L-selectins. This inhibitory mechanism is similar to that described for the anti-inflammatory activities described above (Borsig et al., 2007; Kozlowski et al., 2011). In the recent work of Zhao and coworkers, the investigators have additionally demonstrated that the sea-cucumber FucCS also inhibits metastasis by targeting the nuclear factor-κ B pathway in melanoma B16F10 cells (Zhao et al., 2013).

### Other activities

Besides coagulation, inflammation, and tumor angiogenesis, the MSPs can also show therapeutic actions in other systems. They can act like wound healing (O'Leary et al., 2004), oxidative-stress (Dore et al., 2013), nociception (Rodrigues et al., 2012), and viral infections (Ponce et al., 2003). In wound healing a combination of chitosan-fucoidan hydrogels were created for therapeutic purposes (Sezer et al., 2008). Nevertheless, the mechanisms of action of MSPs in these systems are yet unclear. However, it is strongly believed that for antiviral activity the MSPs might be impairing the adhesion of the virus particle onto host cells since many virus need the negatively charged polysaccharides on host cell surfaces for attachment and invasion. The clinical systems just described here comprise new research areas for MSPs in terms of studying their underlying mechanisms of action and the structural features necessary for the effectiveness.

## Remarked conclusions

Here, we have made clear the clinical significance of MSPs. Chitin and chitosan (Figure 1A) are likely the mostly abundant polysaccharides from the marine environment. They can show beneficial effects in immune response, against cancer, in hemostasis, as hypocholesteromic and hypolipidemic agents besides exhibiting capacity to enhance drug delivery. Even though they exhibit an impressive range of therapeutic actions, chitin/chitosan fibers have mostly been used in the pharmaceutical market as merely dietary supplements for weight control. GAGs from marine organisms are really distinct in terms of function and structure. Two main examples are the ascidian DS with different patterns of sulfation (Figure 1B) and the sea-cucumber FucCS (Figure 1C). The latter differs considerably from the common CS due to the presence of fucosyl branches. This branch is a structural requirement for the biomedical properties since when it is removed the sea-cucumber SP losses its medical properties. As opposed to CS, FucCS can be used as a potential anti-inflammatory and anticoagulant agent. Both ascidian DS and FucCS have not been employed in researches of clinical trials. They have been used only in *in vitro* and *in vivo* studies. The *in vivo* experiments have mostly used laboratory wild and mutant mice models. SFs and SGs are other important classes of SPs found in the sea. In invertebrates and in some red algae, these compounds may exist with well-defined chemical structures (Table 2). The use of these structurally well-defined glycans has helped the development of drug discovery by achieving accurate structure-function relationships. These unique glycans has also helped to understand the underlying mechanisms of action involved in some clinical effects of the MSPs. The clinical events with mechanisms of action mostly elucidated so far are anti-inflammation, anticoagulation, antithrombosis, and anti-tumor angiogenesis. Although brown algae SFs, widely known as fucoidans, do not have well-defined chemical structures, they are the mostly used MSPs in research. Like chitin and chitosan fibers, the brown algal SFs have been used as dietary supplement products in the market. Clinical trials in animals are likely to be unknown for the majority of the MSPs discussed here. The clinical tests available so far are just those found in the referential works cited through this document.

## Marine medicial glycomics

This document has as its main objective the description of the most important marine carbohydrates with therapeutic actions, as well as their main structural and medical properties. These glycans are really unique, and this uniqueness seems to be related to the marine source. Glycomics, as an area of research, has grown significantly over the last few years. Based on the discoveries made with respect to therapeutic properties of marine glycans, as discussed here, we want to propose to the major international scientific societies involved with drug development, glycobiology, and marine biology, a glycomics subproject named *marine medicinal glycomics*. The subproject *marine medicinal glycomics* would be very useful to push forward the research programs involved with marine carbohydrate-based drug development. Since clinical tests using the marine glycans here discussed, especially those of Table 2, are virtually inexistent, the implementation of this subproject would support research programs of licensed clinical trials using these sugars. The implementation of this subproject would also enhance the medical contribution of carbohydrates in the currently ongoing glycomic age. Not only chitin/chitosan, invertebrate GAGs, SFs, and SGs would benefit from this subproject, but actually, any marine carbohydrate possessed of medical properties. Certainly the number of marine carbohydrate-based drugs would increase significantly with the implementation of such subproject.

### Conflict of interest statement

The author declares that the research was conducted in the absence of any commercial or financial relationships that could be construed as a potential conflict of interest.

## Abbreviations

AMCase: acidic mammalian chitinase
aPTT: activated partial thromboplastin time
AT: antithrombin
bFGF: basic fibroblast growth factor
BCT: blood coagulation time
FGFR: fibroblast growth factor receptor
DS: dermatan sulfate
Fuc*p*: L-fucopyranose
FucCS: fucosylated chondroitin sulfate
GAGs: glycosaminoglycans
GalNAc: N-acetyl D-galactosamine
Gal*p*: galactopyranose
GlcA: D-glucuronic acid
GlcN: D-glucosamine
GlcNAc: N-acetyl D-glucosamine
GnT-V: N-acetylglucosaminyltransferase-V
HCII: heparin cofactor II
HMWC: high molecular weight chitosans
ICAM: intercellular cellular adhesion molecule
IdoA: L-iduronic acid
LMWC: low molecular weight chitosans
Man: mannose
MMWC: medium molecular weight chitosans
MSPs: marine sulfated polysaccharides
MW: molecular weight
PA: platelet aggregation
PRP: platelet-rich plasma
PDGF-AB: platelet derived growth factor-AB
PSGL-1: P-selectin glycoprotein ligand-1
RANTES: regulated on activation normal T-cell expressed and secreted
SGs: sulfated galactans
SFs: sulfated fucans
SPs: sulfated polysaccharides
TGF-β: transforming growth factor-β
VEGF: vascular endothelial growth factor
IIa: thrombin
Xa: factor X activated
XIIa: factor XII activated.
